# A selective retinoid X receptor agonist bexarotene (LGD1069, targretin) inhibits angiogenesis and metastasis in solid tumours

**DOI:** 10.1038/sj.bjc.6602995

**Published:** 2006-02-21

**Authors:** W-C Yen, R Y Prudente, M R Corpuz, A Negro-Vilar, W W Lamph

**Affiliations:** 1 Department of Molecular Oncology, Ligand Pharmaceuticals, Inc., San Diego, CA 92121, USA

**Keywords:** bexarotene, retinoid X receptor, angiogenesis, metastasis

## Abstract

The present study determined the influence of a retinoid X receptor agonist bexarotene on angiogenesis and metastasis in solid tumours. In the experimental lung metastasis xenograft models, treatment with bexarotene inhibited the development of the lung tumour nodule formation compared to control. *In vivo* angiogenesis assay utilising gelfoam sponges, bexarotene reduced angiogenesis in sponges containing vascular endothelial growth factor, epidermal growth factor and basic fibroblast growth factor to various extent. To determine the basis of these observations, human breast and non-small-cell lung cancer cells were subjected to migration and invasion assays in the presence of bexarotene. Our data showed that bexarotene decrease migration and invasiveness of tumour cells in a dose-dependent manner. Furthermore, bexarotene inhibited angiogenesis by directly inhibiting human umbilical vein endothelial cell growth and indirectly inhibiting tumour cell-mediated migration of human umbilical vein endothelial cells through Matrigel matrix. Analysis of tumour-conditioned medium indicated that bexarotene decreased the secretion of angiogenic factors and matrix metalloproteinases and increased the tissue inhibitor of matrix metalloproteinases. The ability of bexarotene to inhibit angiogenesis and metastasis was dependent on activation of its heterodimerisation partner peroxisome proliferator-activated receptor *γ*. Collectively, our results suggest a role of bexarotene in treatment of angiogenesis and metastasis in solid tumours.

Metastasis represents a major challenge in the therapeutic management of cancer patients. About 60% of patients have microscopic or clinically evident metastases at the time of diagnosis of their primary tumours (review in Fidler, 2000a). Currently, systemic chemotherapy is the major modality in treatment of metastatic diseases; however, this treatment is usually palliative but not curative in part due to poor response of metastatic tumours to chemotherapy (Fidler *et al*, 1994; Song *et al*, 2000). Thus, identification of effective treatment regimens for metastasis is urgently needed.

Over the past decade, research effort on metastatic disease has been focused on the biological processes that influence the establishment of metastases. It has been well established that tumour metastasis is a complex multistep process that requires migration, invasion and angiogenesis (Fidler, 2000a). The development of adequate blood supply, or angiogenesis, is an important initial step for the growth and spread of a solid tumour (Folkman, 1985). Angiogenesis begins with an angiogenic stimulus followed by local degradation of the basement membrane surrounding the capillaries. A number of angiogenic stimulators including vascular endothelial growth factors (VEGF), basic fibroblast growth factor (bFGF), epidermal growth factor (EGF), platelet-derived endothelial cell growth factor (PDGF) were identified to stimulate endothelial cell proliferation resulting in angiogenesis *in vivo* (Kumar and Fidler, 1998). Importantly, the expression of some angiogenic factors, notably VEGF and bFGF, has been shown to correlate directly with the metastatic potential in solid tumours (Fidler, 2000a). In addition to angiogenesis, interaction of tumour cells with the extracellular matrix (ECM) is a crucial step in the metastatic cascade. These processes facilitate growth and spread of the tumour cells from the primary site of origin to secondary sites by degrading surrounding ECM and basement membrane. The proteolytic degradation of the basement membranes occurs at multiple stages throughout the invasive and metastatic cascade. Among several proteases, matrix metalloproteases (MMPs) play an important role in this process. The metastatic potential of tumour cells has been shown to be correlated with the expression and activities of MMPs in experimental metastasis models (Stetler-Stevenson, 2001). Both angiogenic factors and MMPs are tightly controlled by their negative regulators. Under normal physiological conditions, negative regulators are dominated. It is believed that the survival and establishment of metastatic lesions depend on a shift in the normal balance of key regulatory factors in favour of invasion and angiogenesis. Thus, control one of these processes represent promising therapeutic targets for cancer therapy.

We previously demonstrated that a selective retinoid X receptor (RXR) agonist bexarotene (LGD1069, Targretin) is an efficacious chemopreventive and chemotherapeutic agent in a number of preclinical rodent models breast cancer (Boehm *et al*, 1994; Gottardis *et al*, 1996; Wu *et al*, 2002a, 2002b). More recently, we have shown that bexarotene in combination with paclitaxel produced a synergistic growth inhibition in a rat carcinogen-induced mammary tumour cell line *in vitro* and resulted in a significant increase in overall objective response compared to single agents alone *in vivo* (Yen *et al*, 2004b). We further demonstrated that the combination of bexarotene/cytotoxic agents prevented and overcame acquired drug resistance in preclinical breast cancer, prostate cancer and non-small cell lung cancer models (Yen *et al*, 2004a; Yen and Lamph, 2005; Yen and Lamph, 2006). Interestingly, cells treated with bexarotene or bexarotene/cytotoxic combination demonstrated reduced invasiveness and angiogenic capacity compared to their drug-resistant counterparts, suggesting that bexarotene may prevent tumour cells from development to more malignant, invasive phenotypes. To explore the role of bexarotene in treatment of solid tumours further, the goal of present study was to evaluate the effect of bexarotene on angiogenesis and metastasis and its potential in treatment of metastatic diseases.

## MATERIALS AND METHODS

### Chemicals and reagents

RPMI 1640 medium, foetal bovine serum, glutamine and gentamycin were obtained from Cambrex Bioscience (Walkersville, MD, USA). Bexarotene was synthesised at Ligand Pharmaceuticals Inc. (San Diego, CA, USA). Polyethylene glycol (Mr 400), Tween 80, carboxymethylcellulose, 3,3′,5,5′, tetramethylbenzidine dihydrochloride (TMB) and 3-(4,5-dimethylthiazol-2-yl)-2,5-diphenyltetrazolum bromide (MTT) were from Sigma Chemicals (St Louis, MO, USA).

### Cell line

Human non-small-cell lung cancer A549 cells and human breast cancer MDA-MB-231 cells were purchased from American Type Culture Collection (Manassas, VA, USA). Both cell lines were routinely cultured in RPMI 1640 supplemented with 10% FBS and 2 mM glutamine. Human umbilical vein endothelial cells (HUVE) were purchased from Cambrex Biosciences (Walkersville, MD, USA) and were maintained in EGM-2 medium supplemented with 5 ng ml^−1^ bFGF, 10 U ml^−1^ heparin, 10 ng ml^−1^ EGF, hydrocortisone, vascular endothelial growth factor, ascorbic acid and 10% FBS.

### *In vivo* experimental metastasis studies

To establish experimental lung metastasis xenograft models, A549 and MDA-MB-231 cells in log phase were harvested and resuspended in sterile saline. At 1 day prior to cell injection, mice were injected intraperitoneally with 0.2 mg of anti-asialo GM1 antibody (Wako Chemicals USA Inc., Richmond, VA, USA) to deplete natural killer cells and macrophages. One million A549 cells or 2 million MDA-MB-231 cells in 200 *μ*l saline were injected through the tail vein of 6-week-old athymic nude mice (Harlan, Madison, WI, USA) with 27-gauge needle. The animals utilised in this study were housed in a United States Department of Agriculture-registered facility in accordance with NIH guidelines for the care and use of laboratory animals. These guidelines met the standards of both the US federal regulations and those required by UKCCCR guidelines for welfare of animals in experimental neoplasia (Workman *et al*, 1998). To determine the effect of bexarotene on inhibition of lung metastasis development, treatment began 1 day after tumour injection. To determine the effect of bexarotene on growth of the existing lung tumours, treatment was initiated 1 week after tumour injection when animals showed at least 5 tumour nodules with 1 mm diameter (determined by visual examination of the lungs of 6–8 randomly selected animals). Each group consisted of 8–9 animals. Bexarotene was suspended in an aqueous solution containing 10% (v v^−1^) polyethylene glycol (Mr 400)/Tween 80 (99.5 : 0.5) and 90% of 1% (w v^−1^) carboxymethylcellulose and dosed orally once daily at 100 mg kg^−1^. This dose of bexarotene was previously determined as the maximum tolerated dose (MTD), the dose that caused <10% weight loss over the course of the study (Gottardis *et al*, 1996). The treatment continued for 6 weeks. Animal weights were recorded once weekly. At the end of study, animals were euthanised, and their lungs were removed, fixed in Bouin's solution (Sigma Chemicals, St Louis, MO, USA). Lung tumour nodules were visualised under a dissecting microscope and the number of tumours was counted.

### *In vivo* angiogenesis assay

To evaluate the effect of bexarotene on angiogenesis *in vivo*, the method of McCarty *et al* (2002) was used with minor modification. Briefly, collagen sponges (1 × 1 × 0.5 mm, Pharmacia and Upjohn, Peapack, NJ, USA) were soaked in sterile saline. Excess saline was removed by blotting. Thereafter, 0.4% agarose containing 1 *μ*g ml^−1^ of VEGF, EGF or bFGF was pipetted onto each sponge. The sponge was allowed to harden prior to implanting subcutaneously into right and left axial regions of 6-week-old male athymic nude mice (Harlan, Madison, WI, USA). Animals were randomised and treated with either vehicle or 100 mg kg^−1^ bexarotene for 4 weeks beginning 1 day or 1 week after sponge implantation. Animals were killed at the end of study, and the sponges were removed and grounded with 0.1 ml sterile double deionised water. Samples were centrifuged and the haemoglobin content was quantified by adding equal amount of TMB to the supernatant. The absorbance was read at wavelength 405 nm. Results were expressed as percent of untreated control.

### *In vitro* drug sensitivity assay

To determine the effect of bexarotene on the growth HUVE cells, the cells were grown in EGM2 growth medium until 80% confluence. Thereafter, cells were trypsinised and seeded in 96-well tissue culture plates in the presence of 1 ng ml^−1^ VEGF, EGF or bFGF in serum-free EGM2 medium overnight. Stock solution of bexarotene was dissolved in DMSO. Sufficient volumes of each stock solution were added to the culture medium so that the final concentration of the solvent was <0.1%. The cells were exposed to various concentrations of bexarotene for 3 days. Drug-induced growth inhibition was measured by MTT assay as described previously (Yen *et al*, 2004a).

### Preparation of conditioned media

A549 and MDA-MB-231 cells were seeded at 1 × 10^6^ cells in T-75 flasks overnight. Thereafter, the cells were washed twice with phosphate-buffered saline and incubated in serum-free culture medium containing 0.1% bovine serum albumin with or without bexarotene for additional 48 h. Control cells were treated similarly in drug-free, serum-free culture medium. The culture supernatant was centrifuged to remove cellular debris and stored at −20°C until use.

### *In vitro* migration assay

The migration assay was carried out in fibronectin-coated 24-well insert system (BD Biosciences, Bedford, MA, USA). Briefly, 1 × 10^6^ A549 or MDA-MB-231 cells were grown in T-75 flask in the presence or absence of bexarotene for 3 days. Thereafter, cells were harvested and resuspended in RPMI medium containing 0.1% BSA at a final concentration of 1 × 10^5^ cells well^−1^ and incubated for 1 h at 37°C. The lower chambers were filled with culture medium containing 5% FBS as a chemoattractant. Cells were incubated for 18–20 h. Cells that migrated through the filter into the lower wells were quantified by MTT assay. Results were normalised with vehicle-treated cells and expressed as migration index.

### *In vitro* invasion assays

The invasion assay was carried out in a Matrigel-coated 24-well Transwell unit (BD Biosciences, Bedford, MA, USA). Briefly, 1 × 10^6^ cells were seeded in T-75 flask overnight and treated with bexarotene at various concentrations for 3 days as described previously. Thereafter, the cells were washed with phosphate-buffered saline, trypsinised, resuspended in serum-free medium and seeded at 1 × 10^5^ cells in triplicate in the upper chamber of Matrigel-coated Transwells. The lower chambers were filled with culture medium containing 5% FBS as a chemoattractant. Cells were incubated for 24 h. To determine the ability of bexarotene to modulate angiogenesis, HUVE at 5 × 10^4^ cells were seeded into the upper chamber of the Matrigel-coated Transwells. The tumour conditioned media (CM) was used as chemoattractant and added to the lower chambers. The fraction of cells invading into and through the Matrigel matrix after 24 h was quantified by MTT assay. Results of invasiveness and angiogenic potential were normalised with vehicle-treated cells and expressed as invasion index.

### Quantification of angiogenic factors, matrix metalloproteinases and tissue inhibitors of matrix metalloproteinases

Level of angiogenic factors (VEGF, bFGF, EGF), metalloproteinases (MMP2 and MMP9) and tissue inhibitors of matrix metalloproteinases (TIMP1 and TIMP2) in the tumour CM was measured by enzyme-linked immunosorbent assay (ELISA) kits (Oncogene Science, Cambridge, MA, USA) according to the procedures provided by the manufacturer. The values were normalised with the total protein concentration in the condition medium and expressed as fold of the vehicle control.

### Transfection of PPAR*γ* small interfering RNA

Tumour cells were grown to 50% confluence and were transfected with PPAR*γ* small interfering RNA (siRNA) (Dharmacon, Lafayette, CO, USA) using Oligofectamine (Invitrogen, Carlsbad, CA, USA) for 3 days in the presence of 1 *μ*M bexarotene. Thereafter, cells were trypsinised and subject to invasion assay. To determine the effect of tumour conditioned medium on HUVE invasion, tumour cells were transfected with PPAR*γ* siRNA in serum-free medium for 2 days. The supernatant was centrifuged and used as chemoattractant in HUVE invasion. Mock transfection was carried out with buffer alone. The sense and antisense strands of siRNA silencing PPAR gene were 5′-AAGUAACUCUCCUCAAAUAUU-3′ and 5′-PUAUUUGAGGAGAGUUACUUUU-3′. Transfection efficiency was determined using a cytotoxic siRNA (Dharmacon, Lafayette, CO, USA). Reduction of PPAR levels was verified using quantitative RT–PCR. Pilot studies indicated that transfection efficiency was about 80% with about 90% reduction of PPAR expression.

### Data analysis

Dose response curves for growth inhibition were generated and were plotted as a percentage of untreated control. Values of IC_50_ (the drug concentration needed to produce 50% growth inhibition) were determined by nonlinear least square regression (JMP, Cary, NC, USA). Differences in mean values between groups were analysed by unpaired Student's *t*-test with 2-tailed comparison. Multiple comparisons used one-way ANOVA test with *post hoc t*-test comparison. Differences of *P*<0.05 are considered significantly different. Software for statistical analysis was by SigmaStat (SPSS Inc., Chicago, IL, USA).

## RESULTS

### Bexarotene inhibits the development of angiogenesis and metastasis *in vivo*

**Figure 1:**
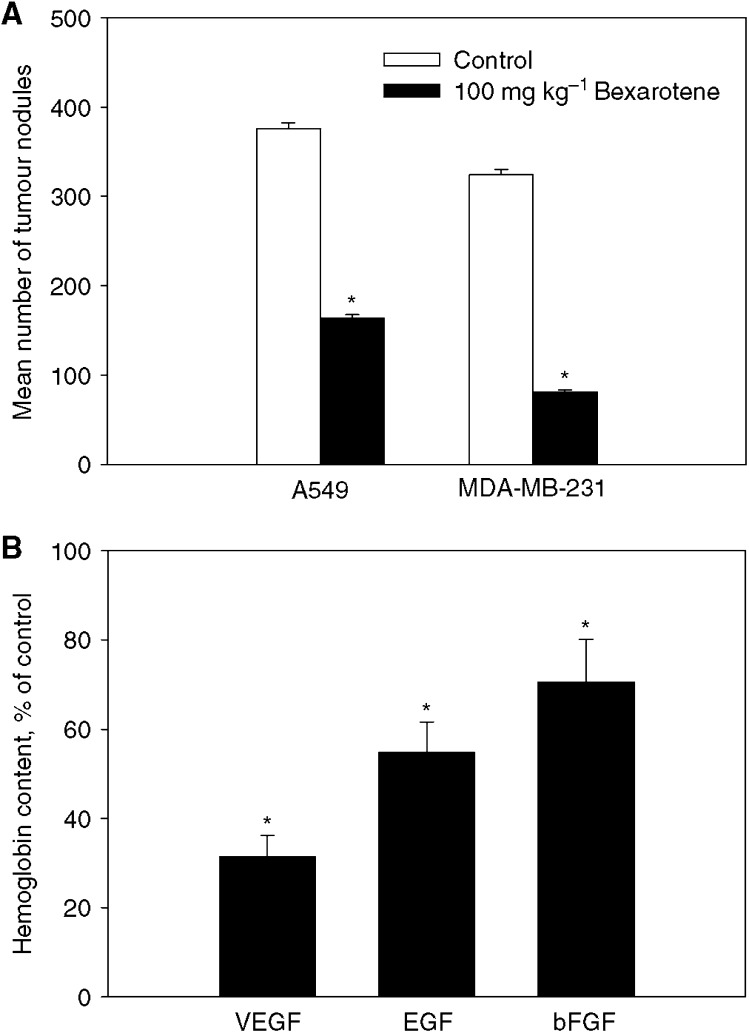
Effect of bexarotene on experimental lung metastasis (**A**) and angiogenesis (**B**) *in vivo*. For experimental metastasis, A549 and MDA-MB-231 cells were injected through tail vein. Animals were randomised and treated with vehicle or 100 mg kg^−1^ bexarotene 1 day after tumour injection for 6 weeks. Drug effect was determined by quantifying numbers of tumour nodules in the lung (Mean±s.e.m., *n=*8–9 animals). For *in vivo* angiogenesis studies, nude mice bearing gelfoam agarose sponges containing proangiogenic molecules were treated with vehicle or bexarotene 1 day after implantation for 4 weeks. Angiogenesis was determined by quantifying haemoglobin content in the sponge and expressed as percent of untreated control (mean±s.e.m., *n=*8 animals). ^*^Statistically different from untreated control at *P*<0.05.

Initial study was to evaluate the role of bexarotene in inhibition of angiogenesis and metastasis *in vivo*. Experimental metastasis models were established in human non-small-cell lung cancer A549 cells and breast cancer MDA-MB-231 cells. As seen in Figure 1A, bexarotene giving alone at 100 mg kg^−1^ daily for 6 weeks reduced more than 50% lung tumour formation compared to the vehicle control in both tumour models when treatment began 1 day after tumour injection. To determine whether bexarotene can influence angiogenesis, gelfoam sponge assay was used to evaluate the efficacy of bexarotene as an antiangiogenesis agent. Vascular endothelial growth factors, EGF and bFGF were used as proangiogenic factors. Blood vessel density was quantified by hemoglobin content. Our data showed that oral daily treatment with bexarotene for 4 weeks significantly reduced proangiogenic factor-mediated angiogenesis, about 70% decrease in blood vessel density against VEGF, 50% against EGF and 30% against bFGF-induced angiogenesis (*P*<0.05 *vs* control in all cases) (Figure 1B). On the other hand, bexarotene had no effect on the growth of existing lung tumours and established blood vessels when treatment began 1 week after tumour injection (data not shown). Taken together, these data demonstrated the ability of bexarotene to inhibit the development of angiogenesis and metastasis *in vivo*. Based on these findings, subsequent studies were conducted to determine factors influencing bexarotene-mediated inhibition of angiogenesis and metastasis.

### Bexarotene inhibits tumour cell migration and invasion *in vitro*

**Figure 2:**
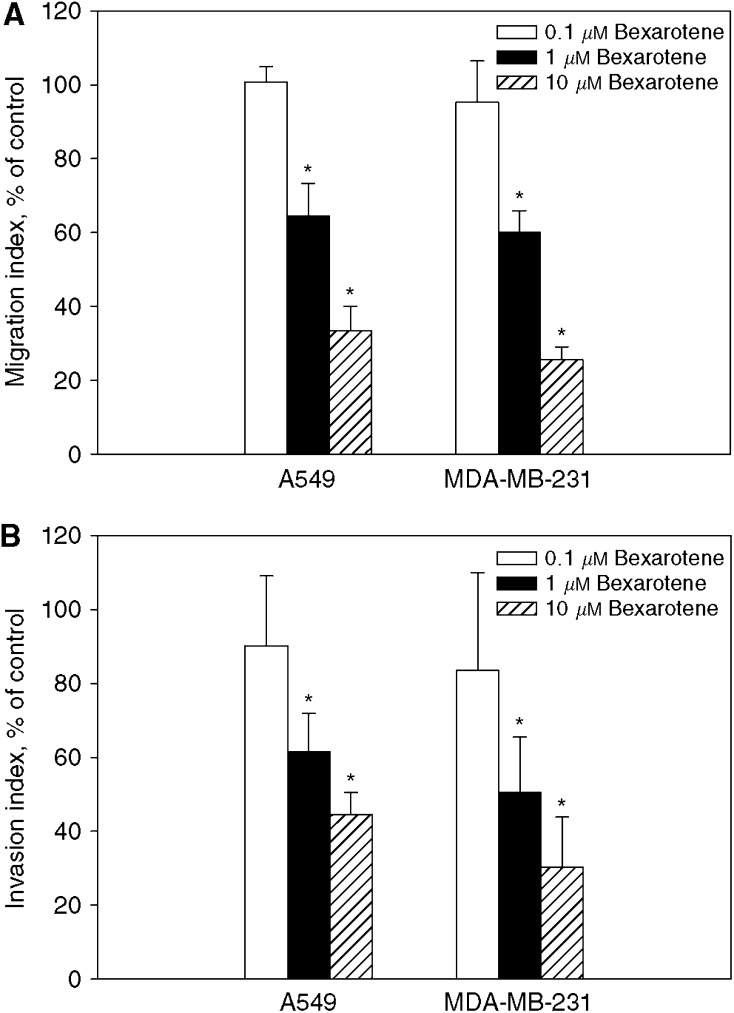
Effect of bexarotene on tumour cell migration (**A**) and invasion (**B**) *in vitro*. A total of 1 × 10^5^ tumour cells were seeded onto the upper chamber of fibronectin coated well for migration assay and the Matrigel-coated Transwells for invasion assay. The lower chambers were filled with culture medium containing 5% FBS as a chemoattractant. The fraction of cells migrating into and through the well was quantified by MTT assay. Results of migration and invasion were normalised with untreated control and expressed as migration and invasion indexes (mean±s.d. from three separate experiments). ^*^Statistically different from untreated control at *P*<0.05.

To determine whether inhibition of lung tumour formation by bexarotene *in vivo* was due to the ability of bexarotene to influence tumour cell migration and/or invasion, the effect of bexarotene on cell migration and invasion was examined. As shown in Figure 2A, migration of A549 and MDA-MB-231 cells were decreased by bexarotene in a dose dependent manner compared to untreated control. Similarly, exposure of cells to bexarotene resulted in inhibition of cell invasion through Matrigel matrix (Figure 2B). The inhibitory effect of bexarotene was not due to cytotoxicity, as neither concentration produced notably effect on cell viability (data not shown).

### Bexarotene has direct and indirect effects on angiogenesis

**Figure 3:**
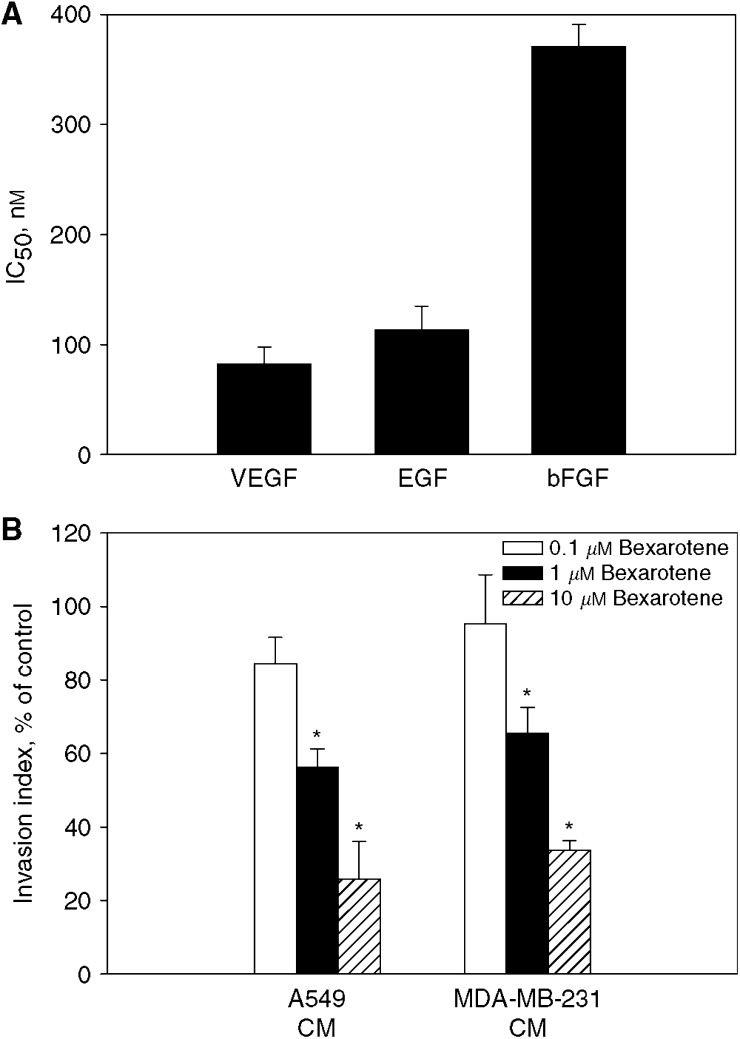
Effect of bexarotene on endothelial cell growth (**A**) and invasion (**B**). For growth inhibition study, HUVE cells were seeded in 96-well tissue culture plates in the presence of 1 ng ml^−1^ VEGF, EGF or bFGF. The effect of bexarotene on proangiogenic factor-stimulated HUVE growth was measured using MTT assay and expressed as IC_50_ (mean±s.d. from three separate experiments). To determine the effect of tumour conditioned medium on HUVE invasion, 5 × 10^4^ HUVE cells were seeded onto the upper chamber of the Matrigel-coated Transwells. The conditioned medium of A549 or MDA-MB-231 cells was served as chemoattractant and added to the lower chambers. The fraction of cells invading into and through the Matrigel matrix was quantified by MTT assay. Results of angiogenic potential were normalised with untreated cells and expressed as invasion index (mean±s.d. from three separate experiments). ^*^Statistically different from untreated control at *P*<0.05.

To determine whether the inhibitory effect of bexarotene on angiogenesis was due to a direct inhibition of endothelial cells, the effect of bexarotene on proangiogenic factor-mediated HUVE cell growth was examined. Bexarotene produced a sigmoidal concentration-dependent decrease in proangiogenic factor-mediated HUVE cell growth, with a maximal inhibition of approximately 90% after 3-day incubation period at 10 *μ*M. The concentration needed to inhibit 50% of cell growth, IC_50_, was 82.8±15.2 nM for VEGF and 137.2±28.2 nM for EGF and 317.1±48 nM for bFGF (mean±s.d., *n=*3, Figure 3A). The growth inhibitory effect of bexarotene on endothelial cells was likely due to inhibition of endothelial cell proliferation since no significant change in apoptosis with bexarotene treatment was observed (data not shown).

It is possible that bexarotene may have an indirect effect on angiogenesis by downregulating tumour cell secreted growth factors. To assess this possibility, the effect of bexarotene on tumour cell conditioned medium-mediated invasion of HUVE cells through Matrigel matrix was evaluated. Our data showed that treatment with 1 *μ*M bexarotene resulted in 50–60% decreased in A549 and MDA-MB-231 conditioned medium-mediated invasion in HUVE cells (Figure 3B). Collectively, these findings indicated that bexarotene inhibited angiogenesis by directly inhibiting human umbilical vein endothelial cell growth and indirectly inhibiting tumour cell-mediated invasion of human umbilical vein endothelial cells through Matrigel matrix.

### Bexarotene inhibits angiogenic factors and MMPs and stimulates TIMPs

**Figure 4:**
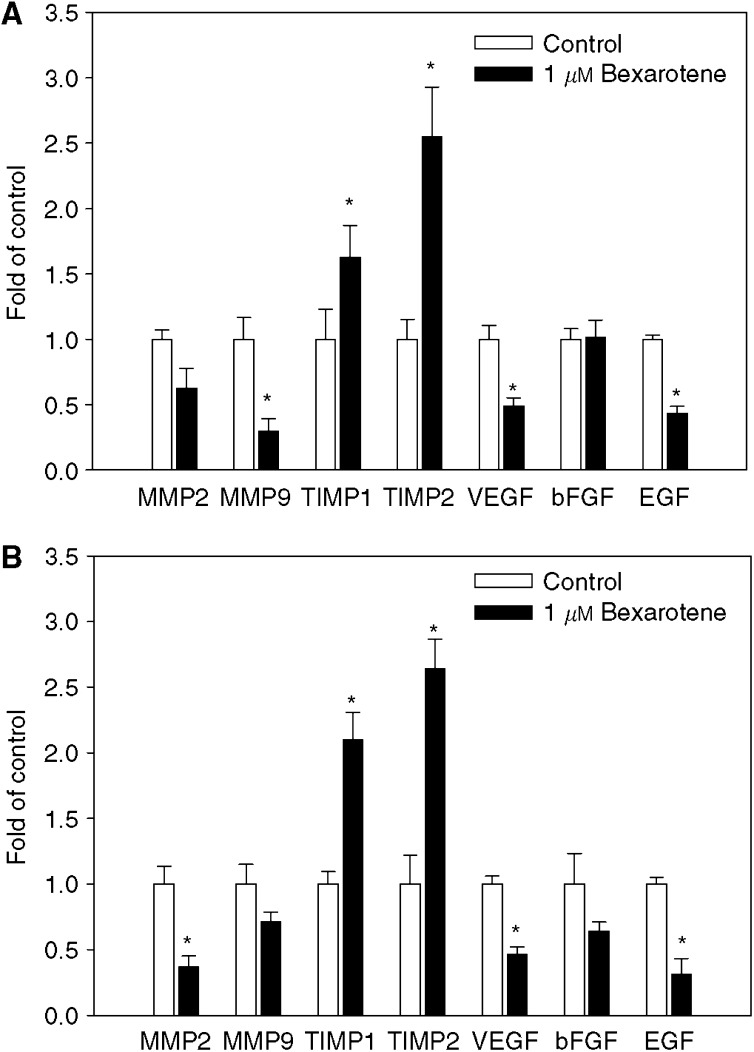
Effect of bexarotene on tumour cell-secreted angiogenic factors, MMPs and TIMPs secretion in A549 cells (**A**) and MDA-MB-231 cells (**B**). The conditioned medium of A549 and MDA-MB-231 cells were prepared as described in Materials and Methods. Levels of MMP2, MMP9, TIMP1, TIMP2, VEGF, EGF and bFGF were analysed by ELISA. The values were normalised with the total protein concentration in the condition medium (mean±s.d. from three separate experiments). ^*^Statistically different from untreated control at *P*<0.05.

To determine whether bexarotene influenced tumour cell-secreted angiogenic factors and metalloproteinases to inhibit invasion and angiogenesis, angiogenic factors, matrix metalloproteinases (MMPs) and tissue inhibitors of matrix metalloproteinases (TIMPs) in tumour-conditioned medium were examined. In A549 cells, treatment with bexarotene resulted in reduction in MMP9, VEGF, EGF and increase in TIMP1 and TIMP2 secretion (Figure 4A). Similar results were observed in MDA-MB-231 cells (Figure 4B). These data indicated that bexarotene-mediated inhibition of invasion and angiogenesis was through regulation of tumour-secreted angiogenic factors, MMPs and TIMPs.

### Loss of PPAR*γ* expression abolishes bexarotene-mediated inhibition of invasion and angiogenesis

**Figure 5:**
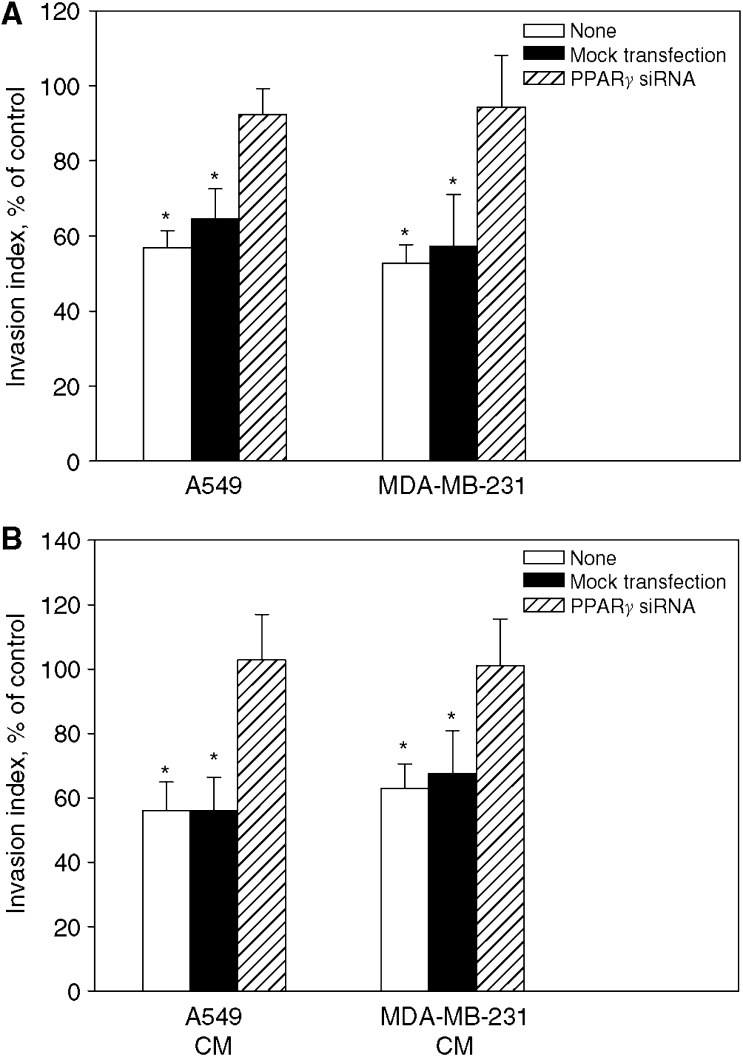
Effect of PPAR*γ* siRNA on bexarotene-mediated inhibition of tumour cells invasion (**A**) and HUVE cell invasion (**B**). Tumour cells were transfected with or without PPAR*γ* siRNA and treated with 1 *μ*M bexarotene for 3 days. Thereafter, tumour cells were subject to invasion assay and conditioned medium were used as chemoattractant for HUVE invasion. The fraction of cells invading into and through the Matrigel matrix was quantified by MTT assay. Results were normalised with untreated cells and expressed as invasion index (mean±s.d. from three separate experiments). ^*^Statistically different from untreated control at *P*<0.05.

To determine whether bexarotene-mediated inhibition of angiogenesis and metastasis was through activation of its heterodimerisation partner peroxisome proliferators activated receptor gamma (PPAR*γ*), PPAR*γ* level in tumour cells were reduced using siRNA. Tumour cells were subject to invasion assay and conditioned medium were used as chemoattractant for HUVE invasion. As seen in Figure 5, similar inhibitory effect by bexarotene was seen in cells without transfection or with mock transfection. On the other hand, bexarotene-mediated inhibition of tumour cell invasion and tumour conditioned medium-mediated HUVE invasion was blocked in the presence of PPAR*γ* siRNA. These data indicated that the inhibitory effect of bexarotene on invasion and angiogenesis was dependent on PPAR*γ* activation.

## DISCUSSION

Metastatic spread is the major cause of cancer death. Current treatments for metastatic diseases are palliative but not curative. Recent advances in tumour biology have increased our understanding in factors responsible for cancer metastasis. It has been recognised that tumour metastasis is a highly selective process. In addition, adequate vascular support is also required for the growth and metastasis of tumour cells beyond 1 mm^3^ in diameter (Folkman, 1985). Thus, targeting against one or more of these processes represent novel approaches for therapeutic management of metastasis.

In the present study, we demonstrated that a selective retinoid X receptor agonist bexarotene inhibited angiogenesis and metastasis in solid tumours. *In vitro* analysis indicated that bexarotene attenuated tumour cell motility and inhibited cell invasion through reconstituted ECM. The ability of bexarotene to interfere with angiogenesis was due to a direct effect on HUVE cell growth and indirect effect on decreasing tumour cell-mediated HUVE cell invasion. Analysis of tumour-conditioned medium demonstrated that bexarotene inhibited angiogenic factors and MMPs and increased TIMPs. The inhibitory effect of bexarotene on angiogenesis and metastasis was through activation of its heterodimerisation partner PPAR*γ*. Collectively, these data suggest a role of bexarotene in treatment of angiogenesis and metastasis in solid tumours.

The growth of both primary and metastatic tumours depends on angiogenesis. The increase in vasculature also increases the ability of tumour cells to invade, enter the circulation to reach distant organs and give rise to a metastasis (Fidler, 2000b). It is proposed that agents that block the molecular events responsible for tumour angiogenesis will be effective against various tumours types. Furthermore, antiangiogenesis approach may avoid the development of acquired drug resistance associated with conventional anticancer therapy since this approach targets genetically stable endothelial cells rather than genetically unstable tumour cells (Boehm *et al*, 1997). Thus, we sought to determine whether bexarotene exerts its antiangiogenic ability in the present study. Our data showed that bexarotene inhibited the development of angiogenesis in sponges containing proangiogenic factors VEGF, bFGF and EGF to various extend *in vivo*. *In vitro* studies indicated that the antiangiogenic effect was due to the ability of bexarotene to inhibit endothelial cell growth and decrease tumour cell-mediated endothelial cell invasion through reduction of VEGF and EGF secretion from tumour cells. In general, the effect of bexarotene was more pronounced against VEGF and EGF-induced angiogenesis and endothelial cell growth compared to its effect against bFGF, suggesting the selectivity of bexarotene against VEGF and EGF-mediated effect. Alternatively, such difference may be due to a greater proliferative stimulation of bFGF on endothelial cells relative to VEGF and EGF.

Migration and local invasion of tumour cells represent initial events in the metastatic processes. Furthermore, tumour invasion requires interaction between the invading tumour cells with the ECM and stromal components. Matrix metalloproteases are key enzymes involved in these processes. Both MMP2 and MMP9, which degrade type IV collagen, have been shown to increase expression in tumour cells and correlated with the malignant phenotype (Stetler-Stevenson, 2001). In the present study, we demonstrated that bexarotene reduced tumour nodule formation in experimental lung metastasis *in vivo* and decrease tumour cell migration and invasion *in vitro*. We further analysed soluble proteins in tumour conditioned medium and found that bexarotene decrease tumour-secreted MMPs and increase TIMPs. MMP2 and TIMP2 were the major secretory proteins found in A549 tumour-conditioned medium, whereas MMP9 and TIMP1 were the key proteinases secreted by MDA-MB-231 cells. Although bexarotene did not significantly change MMP2 secretion in A549 cells and MMP9 in MDA-MB-231 cells, the decreased amount of other matrix metalloproteinases and elevated level of TIMPs may result in an increased ratio of TIMP to MMP leading to a decrease in tumour cell invasion.

PPAR*γ* is a ligand-activated nuclear receptor that is critical in a variety of biological processes (Rosen and Spiegelman, 2001). Activation of PPAR*γ* has been showed to inhibit proliferation and induce apoptosis in a variety of malignant tumours (Kubota *et al*, 1998; Mueller *et al*, 1998; Kitamura *et al*, 1999; Motomura *et al*, 2000; Tsubouchi *et al*, 2000; Ohta *et al*, 2001; Yang and Frucht, 2001). In addition, several reports have indicated the involvement of PPAR*γ* agonists in expression of angiogenic and matrix metalloproteinase factors (Fauconnet *et al*, 2002; Worley *et al*, 2003; Panigrahy *et al*, 2005). Mechanistic studies indicated that activation of PPAR*γ* interferes with the activities of the transcription factors activation protein 1 (AP1) and nuclear factor-*κ*B (NF-*κ*B)(Ricote *et al*, 2000). NF-*κ*B is closely associated with tumour angiogenesis and metastasis (Shishodia and Aggarwal, 2004). As RXR forms heterodimeric complex with PPAR*γ* to activate target gene expression, we hypothesized that bexarotene-mediated inhibition of invasion and angiogenesis was through activation of its heterodimerisation partner PPAR*γ*. This hypothesis was supported by lack of bexarotene-induced inhibitory effect on tumour invasion and tumour conditioned medium-mediated HUVE cell invasion in the presence of PPAR*γ* siRNA.

In summary, we demonstrated that bexarotene inhibited the development of angiogenesis and metastasis *in vivo*. The inhibitory effect of bexarotene was due to its ability to inhibit tumour cell migration and invasion, inhibit endothelial cell growth and interfere with tumour cell-mediated invasion of endothelial cells. These findings have important implications for patients with uncontrolled locoregional diseases. For example, bexarotene can be used alone or incorporated with other conventional modalities such as radiation and chemotherapy to improve the efficacy of these treatments as well as to prevent local recurrence and development of distant metastasis. On-going research is directed towards understanding the mechanism by which bexarotene inhibits angiogenesis and metastasis. Information obtained from these studies will have significant impact on the therapeutic use of bexarotene in treatment of metastatic diseases.
